# Comparative Evaluation of the Cytotoxicity of Doxorubicin in BT-20 Triple-Negative Breast Carcinoma Monolayer and Spheroid Cultures

**DOI:** 10.3390/biomedicines11051484

**Published:** 2023-05-19

**Authors:** Keith N. Ncube, Tamarin Jurgens, Vanessa Steenkamp, Allan D. Cromarty, Iman van den Bout, Werner Cordier

**Affiliations:** 1 Department of Pharmacology, Faculty of Health Sciences, University of Pretoria, Pretoria 0007, South Africa; keith.ncube@up.ac.za (K.N.N.); vanessa.steenkamp@up.ac.za (V.S.); duncan.cromarty@up.ac.za (A.D.C.); 2 Department of Physiology, Faculty of Health Sciences, University of Pretoria, Pretoria 0007, South Africa; tamarinjurgens@gmail.com (T.J.); iman.vandenbout@up.ac.za (I.v.d.B.); 3 Centre for Neuroendocrinology, Department of Immunology, University of Pretoria, Pretoria 0007, South Africa

**Keywords:** two-dimensional culture model, three-dimensional culture model, BT-20 cells, doxorubicin, drug susceptibility, multicellular spheroids, triple-negative breast cancer

## Abstract

Three-dimensional cell culture models are increasingly adopted as preferred pre-clinical drug testing platforms, as they circumvent limitations associated with traditional monolayer cell cultures. However, many of these models are not fully characterized. This study aimed to characterize a BT-20 triple-negative breast carcinoma spheroid model and assess its susceptibility to doxorubicin in comparison to a monolayer model. Spheroids were developed using the liquid overlay method. Phenotypic attributes were analyzed by characterizing changes in size, gross morphology, protein content, metabolic activity, hypoxic status, and cell–cell junctions. The cytotoxic range of doxorubicin in monolayers was determined using the sulforhodamine B assay, and the comparative effect of toxic and sub-toxic concentrations was assessed in both spheroids and monolayers. Similar to the in vivo microenvironment, spheroids had a heterogeneous spatial cytoarchitecture, inherent hypoxia and strong adherens junctions. Doxorubicin induced dose-dependent cytotoxicity in monolayers (IC_25_: 130 nM, IC_50_: 320 nM and IC_75_: 1580 nM); however, these concentrations did not alter the spheroid size or acid phosphatase activity. Only concentrations ≥6 µM had any effect on spheroid integrity. In comparison to monolayers, the BT-20 spheroid model has decreased sensitivity to doxorubicin and could serve as a better model for susceptibility testing in triple-negative breast cancer.

## 1. Introduction

Breast cancer is the most frequently diagnosed cancer in women and the leading cause of cancer mortality globally [1]. Approximately 12 to 17% of breast cancer patients are diagnosed with triple-negative breast cancer (TNBC), a subtype that has low expression of HER-2/ERBB2 and lacks both estrogen and progesterone receptors [2]. A concerning trait of TNBC is its aggressive nature and high relapse rates, compared to non-TNBCs [2]. Currently, there is a lack of effective, specific therapies for TNBC, due to the inability to target a specific molecular characteristic or receptor [3].

Third-generation adjuvant or neoadjuvant regimens currently used to treat TNBC, consist of a taxane combined with an anthracycline/cyclophosphamide-based formulation [2]; however, this approach occasionally fails to slow tumor progression [4]. Despite the advancement of genetic and molecular drivers for characterization of cancer, oncology drugs are associated with higher attrition rates compared to drugs for other ailments [5]. Though there are various reasons for these high attrition rates, the historic inability to efficiently translate pre-clinical research to clinical success is a major contributor [6]. It is therefore imperative that the experimental cellular environment in in vitro pre-clinical drug screenings closely represent the phenotype of cells within the in vivo target tissue [7].

Many research groups in academia and the biomedical industry use two-dimensional (two-dimensional) cell culture models for pre-clinical drug screening [8]. Inadequate in vivo tumor mimicry of these traditional two-dimensional models results in the poor translation of therapeutic outcomes for in vivo models [9]. Animal models are proven to provide a more accurate representation of the tumor environment; however, they are time consuming, less reproducible and expensive to perform, compared to cell cultures employed in a high-throughput drug screening [10]. In a bid to circumvent these experimental inconsistencies, three-dimensional (three-dimensional) cell culture models have been suggested as a potential bridge between in vitro two-dimensional models and animal models [11].

Multicellular spheroids are a classic example of a three-dimensional cell culture model. Spheroids are heterogeneous cellular aggregates that resemble avascular tumor nodules/micro metastases or intervascular regions of large solid tumors with respect to microenvironmental cues, gene expression profiles, growth kinetics and some histomorphological features [12].

The attainment of optimal correlation with such in vivo phenotypic attributes relies on fit-to-purpose culture conditions. Therefore, prior to use in pre-clinical research, spheroid models should be extensively characterized to best understand in vitro phenotype dynamics. Although multicellular spheroids have been used for in vitro simulation of in vivo micromilieu [13], their phenotypic properties have not been widely investigated [14]. Elucidation of structural and functional characteristics of spheroids could potentially reveal inherent parameters that could be targeted by new test compounds. Additionally, well-characterized, three-dimensional cultures could be routinely adapted as ‘spheroid controls’ or standards, when conducting drug screening on poorly characterized spheroid types [12]. Therefore, this study aimed to elucidate some of the histomorphological characteristics of a spheroid model of the BT-20 TNBC cell line during growth, and compare the cytotoxicity of doxorubicin in the spheroids’ and monolayers’ counterparts.

## 2. Materials and Methods

### 2.1. Cell Culture and Maintenance of the BT-20 Cell Line

The TNBC BT-20 (ATCC^®^ HTB-19^TM^) cell line was grown in T75 flasks (Corning^®^ CellBIND^®^, Corning, NY, USA) using a mixture (1:1) of Dulbecco’s Modified Essential Medium (DMEM) and Ham’s-F12 medium (Gibco-Life Technologies, Waltham, MA, USA). This medium was supplemented with 10% heat-inactivated foetal bovine serum (FBS; Gibco-Life Technologies, Waltham, MA, USA) and 1% penicillin–streptomycin (Gibco-Life Technologies, Waltham, MA, USA). Cell culture conditions were maintained at 5% carbon dioxide (CO_2_) at 37 °C in a humidified environment. Cells were grown to 80% confluence, rinsed with phosphate-buffered saline (PBS), detached using Trypsin/Versene digestion, and diluted to 5 × 10^5^ cells/mL.

### 2.2. Formation of Multicellular Spheroids

Spheroids were generated using the agarose-based liquid overlay method described by Friedrich et al. [12] with optimization of culturing medium and seeding density [15]. Agarose (1% *w*/*v*) was prepared in FBS-free DMEM: Ham’s-F12 medium (1:1) and solubilized by sterilization at 120 °C for 30 min in an autoclave, then stored at 4 °C. Corning^®^ 96-well microtiter plates were then treated with pre-heated agarose (50 µL) to create a low-attachment surface. Plates were cooled to room temperature in a sterile environment until the agarose in the wells solidified. Cells (100 µL, 5 × 10^4^ cells/well) were seeded into the agarose-coated microtiter plate, and an additional 100 µL 10% FBS-fortified medium added. Plates were incubated for ten days. Medium (100 µL, 10% FBS) was replaced after four days and thereafter every two to three days.

### 2.3. Characterization of Spheroids

#### 2.3.1. Size and Morphology of Spheroids

To determine the change in spheroid size and morphology, images of all spheroids were taken at Day 4, 7 and 10 using a Zeiss Axiovert 200 M inverted microscope with an attached Zeiss AxioCam MRm 1.4-megapixel CCD monochrome microscope camera with a 1388 × 1040 resolution (Carl Zeiss Inc., Oberkochen, Germany) using a 5× objective lens. Images were saved as .zvi files, then an ImageJ-based, automated spheroid size measurement algorithm [16] was used to calculate the volume of the spheroids.

#### 2.3.2. Change in Spheroid Protein Content

The protein content per spheroid (Day 4, 7 and 10) was determined using the bicinchoninic acid (BCA) assay [17]. Eight spheroids (Day 4, 7 and 10) were pooled and washed twice with PBS (200× *g* for 5 min). Thereafter, the spheroids were lysed using a 100 μL radioimmunoprecipitation assay buffer, prepared in-house: (50 mM Tris-hydrochloride [pH 7.4], 150 mM sodium chloride, 1% Triton X-100, 1% sodium deoxycholate, 0.1% *w*/*v* sodium dodecyl sulfate, 1 mM EDTA and 0.02% *w*/*v* Roche cOmplete^TM^ protease inhibitor cocktail), then centrifuged for 10 min at 16,000× *g*, and the supernatant lysate used for protein determination. Into a clear, flat-bottom 96-well plate, 5 μL bovine serum albumin (BSA) standards (0.1, 0.2, 0.25, 0.5, 1.0, 1.5 and 2.0 mg/mL) or spheroid lysate, and 195 μL BCA reagent was added. The plate was shaken for 10 min at room temperature, then incubated at 60 °C for 30 min. The plate was cooled to room temperature and the absorbance measured with an ELX800UV microplate reader (BioTek instruments Inc., Highland Park, IL, USA), using a 570 nm flitter with a 10 nm band width.

#### 2.3.3. Live/Dead Status Using the Fluorescein Diacetate Propidium Iodide Staining Protocol

Live/dead staining [18] was conducted to evaluate the viability of the spheroids. Harvested spheroids were washed three times with 0.1 M PBS, then transferred to a Corning^®^ 24-well plate. Spheroids were stained for 4 min in the dark using a staining solution (1 mL) consisting of 4 µg/mL propidium iodide (PI; Sigma Aldrich, St Louis, MO, USA) and 5 µg/mL fluorescein diacetate (FDA; Sigma Aldrich, St Louis, MO, USA) in PBS. The excess staining solution was removed by washing three times with 1 mL PBS (1 min), and the spheroids were immediately visualized with a Zeiss Axiovert 200 M inverted microscope (Carl Zeiss Inc., Oberkochen, Germany) with filter sets for Texas red (PI) and fluorescein (FDA) fluorescence, using a 5× objective lens. Images were captured using a Zeiss AxioCam MRm 1.4-megapixel CCD monochrome microscope camera with a 1388 × 1040 resolution (Carl Zeiss Inc., Oberkochen, Germany). ImageJ (version 1.52k) was used to create a composite of images taken under the different filters.

#### 2.3.4. Histological Analysis of the Spheroids

Histological analysis was performed using the hematoxylin and eosin-staining (H&E) method [19]. Spheroids (Day 4, 7 and 10) were harvested and fixed in 4% *v*/*v* formalin in 0.1 M PBS, sequentially dehydrated manually using ethanol, and embedded in paraffin wax. Embedded spheroids were sectioned into 5 µm sections using a Leica RM2255 microtome (Lecia Microsystems, Wetzlar, Germany) and mounted on microscope slides. The slides were deparaffinized with xylene, then sequentially rehydrated with a final wash in distilled water for 1 min. Slides were stained with hematoxylin (0.1% *w*/*v* for 10 min), rinsed with Scott’s buffer for 10 min, dipped in eosin (2% *w*/*v*) for 2 min, and rinsed with distilled water for 1 min. Sections were dehydrated then sealed with a cover slide using a xylene-based mounting media. These mounted sections were observed under an Olympus IX71 inverted microscope (Olympus Corporation Shinjuku, Tokyo, Japan), ZEISS Axiocam ERc 5s 5-megapixel microscope camera with a 2560 × 1920 resolution, using a 10× and 40× objective lens. Images were saved as .zvi files and processed using ImageJ (version 1.52k).

#### 2.3.5. Cadherin Staining of Spheroids

Prior to cadherin staining, whole spheroids were cleared using the T2 clearing protocol, as described by Kuwajima et al. [20]. After clearing, spheroids were permeabilized using 0.1% Triton X-100 in PBS. Permeabilized spheroids were blocked in 2% BSA in PBS, before they were exposed to a mouse anti-pan-cadherin monoclonal antibody (Sigma Aldrich, St Louis, MO, USA). After washing, spheroids were exposed to 4′,6-diamidino-2-phenylindole (DAPI) and Alexa Fluor 488-conjugated donkey anti-mouse secondary antibody (Sigma Aldrich, St Louis, MO, USA). Spheroids were then mounted in the ProLong™ Glass Antifade Mountant (ThermoFisher Scientific, Waltham, MA, USA) under a coverslip, and imaged using a Zeiss LSM800 confocal microscope. Images were generated using a 20× air objective, resulting in images at a resolution of 0.217 µm/pixel. Z-stacks were acquired every 2 µm. Nuclear staining (via DAPI) was visualized by excitation at 405 nm and emission detected up to 541 nm. Cadherin was visualized through excitation at 488 and emission detected up to 700 nm.

#### 2.3.6. Assessment of the Induction of Hypoxia

On Day 4, 100 µL of medium from the spheroid media was replaced with 10 µM of the Image-iT^®^ hypoxia probe (Invitrogen, Carlsbad, CA, USA), prepared in 10% FBS-supplemented medium to achieve a 5 µM in-well concentration. After 24 h, the fluorescence of the probe was imaged with a Zeiss Axiovert 200 M inverted microscope with an attached Zeiss AxioCam MRm 1.4-megapixel CCD monochrome microscope camera with a 1388 × 1040 resolution (Carl Zeiss Inc., Oberkochen, Germany), using a Texas red filter set at 5× objective magnification. Images were transferred and analyzed using ImageJ (version 1.52k).

### 2.4. Elucidation of the Cytotoxic Concentrations of Doxorubicin in Monolayers

Attached BT-20 monolayer cultures were exposed to 100 μL medium (negative control), saponin (1%, positive control), dimethyl sulfoxide (DMSO; 0.2%, vehicle control) or half-log dilutions of doxorubicin (highest concentration of 32 μM) prepared in FBS-free medium for 72 h. A sterility and background noise control consisted of 200 μL medium supplemented with 5% FBS. After exposure, cell enumeration was determined using the sulphorhodamine B (SRB) protocol of Vichai and Kirtikara [21].

#### Comparison of the Cytotoxicity of Doxorubicin in Monolayers and Spheroids

For the two-dimensional monolayer culture model, 100 μL medium (negative control), DMSO (0.2%, vehicle control), saponin (1%, positive control), or sub-toxic and toxic concentrations of doxorubicin (IC_25_, IC_50_ and IC_75_, in-reaction) prepared in FBS-free medium, were added to attached cells. Spheroids were grown for four days using the liquid overlay method, and 100 µL medium replaced with two-fold IC_25_, IC_50_, and IC_75_ concentrations as determined from monolayer cultures, as well as higher concentrations (6 µM, 8 µM and 10 µM in-well) due to hypothesized reduced drug sensitivity. The effect of doxorubicin on both models was assessed after 72 h using the acid phosphatase (APH) assay [22] and through visualization of morphological changes.

### 2.5. Statistics

Microsoft Excel 2019 (Microsoft) was used to capture raw data and statistical analyses performed with GraphPad Prism 8.0.2 (GraphPad Software, San Diego, CA, USA). At least three biological repeats were conducted per experiment, with four technical repeats for monolayer studies, and at least six representative spheroids per experimental condition. All data was expressed as the mean and standard error of the mean (SEM). Changes in spheroid protein content and volumes over the culturing period were calculated using a Kruskal–Wallis analysis of variance (ANOVA) test with a Dunn’s multiple comparison post-test. For monolayer cytotoxicity studies, the logarithmic drug concentration was plotted against the relative response (compared to the negative control). The cytotoxic range of doxorubicin was calculated using a non-linear regression curve fit (log[doxorubicin] vs. relative cell number) with a robust fit. A comparison of the changes in spheroid viability and volume after exposure to the different concentrations of doxorubicin was determined using a Kruskal–Wallis test with a Dunn’s multiple comparison post-test (*p* > 0.05).

## 3. Results

### 3.1. Spheroids Compact over the Growth Period

**Figure 1:**
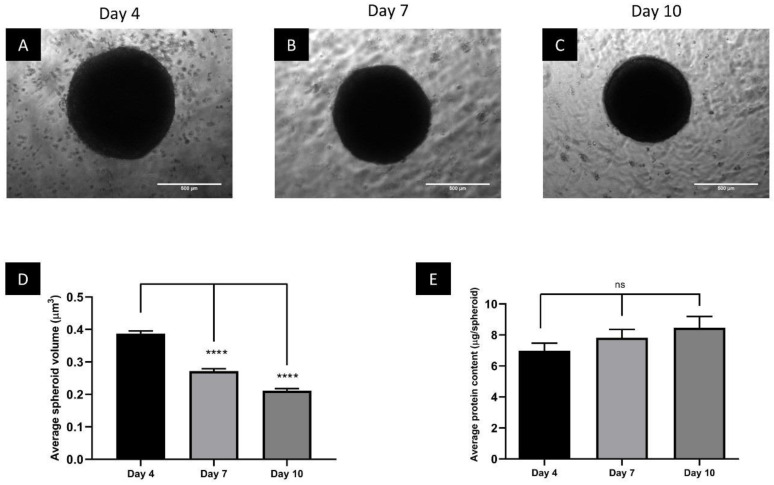
Phase contrast images of spheroids grown using the liquid overlay method on Day 4 (**A**), Day 7 (**B**) and Day 10 (**C**). Images captured using a 5× objective lens, scale bar = 500 µm. Graphs showing the change in average spheroid volume (µm^3^) (**D**) and protein content per spheroid (µg/spheroid) (**E**). ns = not significant. **** = *p* ≤ 0.0001.

The stability of spheroids over time was determined to assess if BT-20 spheroids could be useful for in vitro chemoresistance in TNBC. By Day 4, spheroids had a circular structure, which was maintained up to Day 10 (Figure 1A–C). The circularity index of 49 randomly selected Day 4 spheroids was 0.87 ± 0.01 µm, suggesting that spheroids were reproducibly spherical.

Spheroid volume was calculated between Day 4 and Day 10 by measuring the diameters to inspect the level of compaction. The average spheroid volume decreased significantly (*p* < 0.0001) from Day 4 (3.9 ± 0.1 × 10^8^ µm^3^) to Day 7 (2.7 ± 0.1 × 10^8^ µm^3^; 31% reduction) and significantly decreased even further (*p* < 0.0001) to Day 10 (2.1 ± 0.1 × 10^8^ µm^3^; 22% reduction) (Figure 1D).

To verify whether the compaction of the spheroids observed through the volume calculations correlated with a reduction in cell number, protein content was used as a proxy. Protein content non-significantly increased between Day 4 (7.0 ± 0.48 µg/spheroid) and Day 7 (7.8 ± 0.54 µg/spheroid), and between Day 7 and Day 10 (8.5 ± 0.73 µg/spheroid) (Figure 1E), representing a 21.4% change from Day 4 to Day 10. The lack of significant change in protein content during the growth period suggests that cell numbers remained relatively constant over the time analyzed, and that the decrease in spheroid volume over time was likely due to compaction rather than cell death.

### 3.2. Spheroids Exhibit a Heterogeneous Architecture Containing a Hypoxic Necrotic Core

**Figure 2:**
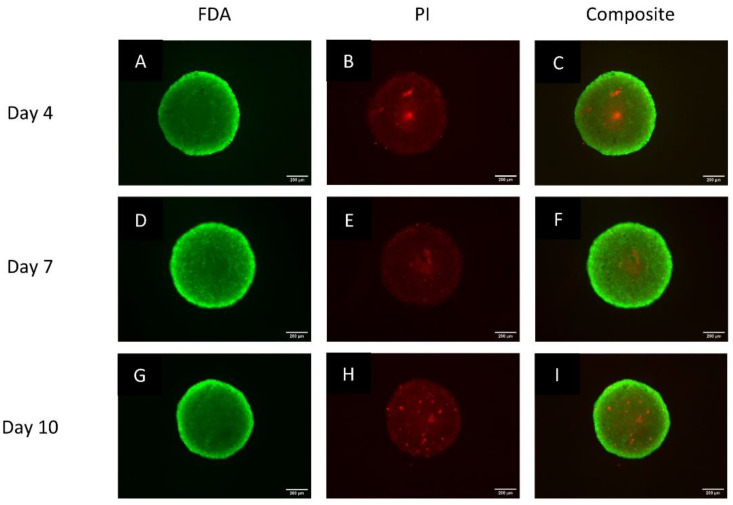
Fluorescence microscopy images of typical spheroids stained with both FDA and PI, and a composite thereof on Day 4 (**A**–**C**), Day 7 (**D**–**F**) and Day 10 (**G**–**I**). Images captured using a 5× objective lens. Scale bar = 200 µm.

To assess the viability of cells over time, the spheroids were stained with the FDA and PI dyes. Most cells within spheroids were viable at all time points, as indicated by the dominance of green fluorescence after FDA staining. However, at Day 4 and Day 7, there was a clearly demarcated inner core where staining was much lower and undefined (Figure 2A–F). This same area tested positive with PI. At Day 10, such a pronounced FDA-negative and PI-positive region seemed dispersed. The fluorescence intensity of FDA was high in cells on the outer spheroid rim, gradually decreasing towards the center. The PI fluorescence was higher in cells situated in the middle region of the spheroids compared to those towards the periphery. The staining of PI was maintained during the growth period; however, on Day 10, the dye was more dispersed compared to Day 4. Therefore, the spheroids remained viable over a period of 10 days with an indication of possible necrosis within inner spheroid regions.

**Figure 3:**
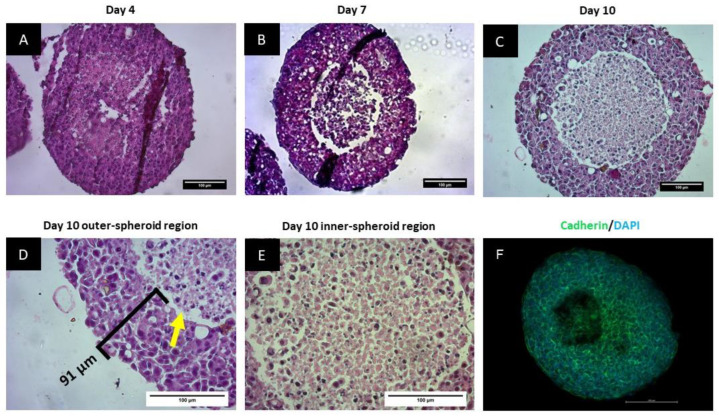
Brightfield images of spheroids stained with H&E on Day 4 (**A**), Day 7 (**B**), and Day 10 (**C**). Spheroids captured using a 20× objective lens. Panels D and E show brightfield micrographs of H&E-stained Day 10 spheroids taken using a 40× objective lens, additionally showing the cellular architecture in the outer (**D**) and inner (**E**) spheroid regions. A distinct gap is visible between the cell layers as shown by the yellow arrow in (**D**). (**F**) shows a composite confocal microscopy image of a spheroid stained with an anti-cadherin antibody (green) and DAPI (blue). Slice 19 of the stack is represented in the image after deconvolution using the default settings in Zen Blue. This image was acquired at approximately 38 µm from the top and 22 µm from the bottom of the spheroid. Scale bars = 100 µm.

Spheroids were sectioned and stained with H&E to elucidate internal cytoarchitecture and to probe the existence of a central necrotic core. The H&E staining pattern was almost homogeneous in Day 4 spheroids with slightly more nuclear (hematoxylin) staining on the outer spheroid regions compared to the inner areas (Figure 3A). The cells in the inner portion of the spheroid were fragmented and smaller with varying sizes of nuclei compared to those on the periphery, suggesting continued spatial differentiation of cellular architecture from Day 4 to Day 7. This differentiation was more pronounced on Day 10, where distinct spatial heterogeneity of cell morphology across the spheroid was evident (Figure 3C). Micrographs of Day 10 spheroids indicated that the outer ~90 µm of the spheroid consisted of densely packed cells with evident staining for both nuclear and cytoplasmic components (Figure 3D). There was a distinct gap separating a cluster of cells in the middle region from the outer rim (yellow arrow in Figure 3D). In contrast, cells in the inner region were smaller, more dispersed, and predominantly stained only with eosin with minimal binding of hematoxylin (Figure 3E). Therefore, the spheroids had a heterogeneous architecture with larger cells on the peripheral rim, and an internal core of possibly necrotic cells. This heterogeneity progressed from Day 4 up to Day 10, when it was more pronounced.

To determine how BT-20 cells aggregate to form spheroids, the cell–cell junctions were visualized after tissue clearing for cadherin and nuclear staining. Figure 3F shows well-developed adherens junctions around all cells. However, a core devoid of nuclei and of cadherin was visible. This data suggests the core of the spheroid to be either empty or only containing cell material that no longer harbored DNA or cadherin protein, consistent with the observations presented with H&E staining.

**Figure 4:**
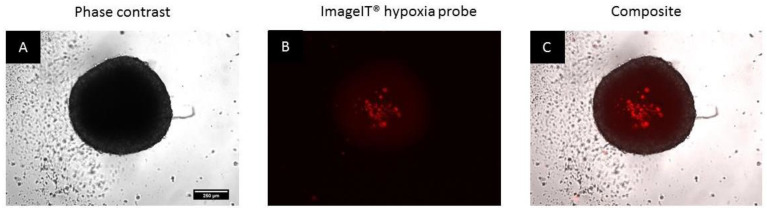
Phase contrast, fluorescence and a composite of spheroids stained with the Image-iT^®^ hypoxia probe on Day 4 (**A**–**C**). Scale bar = 250 µm.

Day 4 spheroids were stained with an Image-iT^®^ hypoxia probe to investigate the induction of hypoxia in spheroid cultures under normoxic conditions. Fluorescence of the Image-iT^®^ red probe was confined to the inner spheroid regions, while the fluorescent signal was slightly attenuated in the outer regions (Figure 4A–C). These observations are suggestive of the inherent development of hypoxia within inner regions of the spheroids leading to the formation of the necrotic core.

### 3.3. The Cytotoxic Efficacy of Doxorubicin Was Reduced in Spheroids Compared to Monolayers

**Figure 5:**
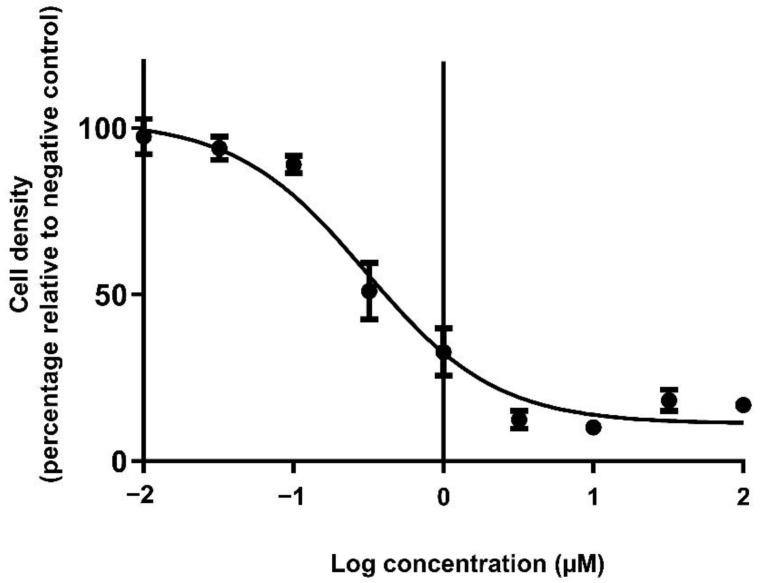
Dose–response curve showing the dose-dependent cytotoxicity of doxorubicin after 72 h exposure. N = 9.

The in vitro cytotoxic efficacy of doxorubicin was compared in monolayers and spheroids. Prior to comparison, the inhibitory concentration (IC) values for doxorubicin on monolayer cultures were determined following 72 h exposure. Untreated- and vehicle-treated cells showed similar cell numbers. Increasing concentrations of doxorubicin resulted in a sigmoidal reduction of cell density (Figure 5). From the dose–response curve, the IC_25_, IC_50_ and IC_75_ values of doxorubicin against the BT-20 monolayer cells were calculated to be 130, 310 and 1580 nM, respectively. The APH viability assay was conducted in parallel as a means of confirming the dose–response observed with the SRB assay, and a similar IC_50_ (278 nM) was obtained (Supplementary File: Figure S1).

**Figure 6:**
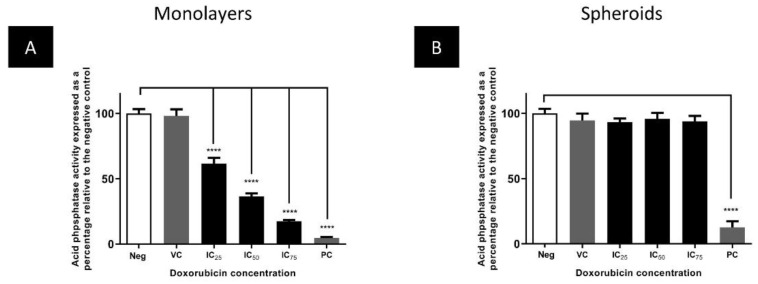
The effect of the IC_25_, IC_50_, and IC_75_ concentration of doxorubicin on the APH activity of monolayers (**A**), and Day 4 spheroids (**B**). **** = *p* ≤ 0.0001. Neg = negative control, PC = positive control, VC = vehicle control.

To directly compare doxorubicin’s cytotoxicity on monolayers and spheroids, the cell numbers were evaluated using the APH assay and morphological changes. In monolayers, APH activity was significantly decreased (*p* ≤ 0.0001) in the monolayers after doxorubicin exposure (Figure 6A). No effect was observed in the spheroid cultures at the monolayer IC_25_ to IC_75_ after 72 h (Figure 6B).

**Figure 7:**
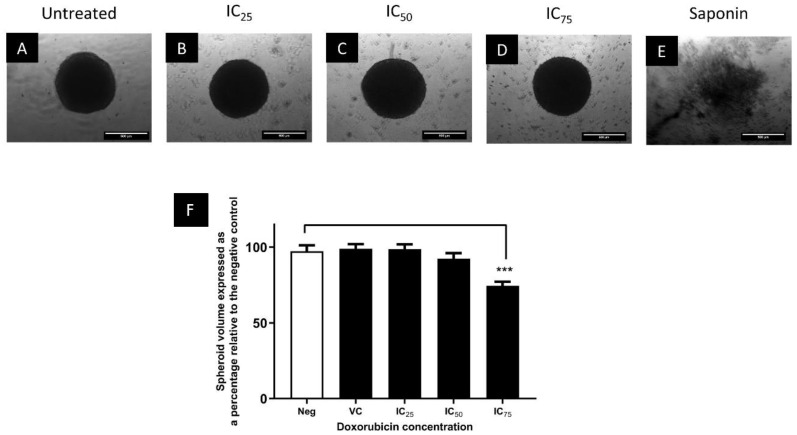
Phase contrast images showing the change in morphology of Day 4 spheroids after 72 h treatment with the negative control (**A**), the monolayer IC_25_ (**B**), IC_50_ (**C**) and IC_75_ (**D**) of doxorubicin and 1% saponin (**E**). Images were captured using a 5× objective lens. Scale bar = 500 µm. Panel (**F**) shows the effect of these concentrations of doxorubicin on the volume of spheroids. *** = *p* ≤ 0.001. Neg = negative control, VC = vehicle control.

Microscopic analysis of spheroids indicated that the morphology and integrity of the spheroids were maintained at 130, 310, and 1580 nM doxorubicin (Figure 7B–D). Spheroid volume was not significantly altered at 130 and 310 nM doxorubicin exposure; however, a significant (*p* ≤ 0.001) 5% reduction in volume was observed at 1580 nM (Figure 7F). Overall, these results indicate that concentrations of doxorubicin that elicit cytotoxicity in monolayer cultures do not significantly alter spheroid volume nor metabolic activity, and support the observation of reduced susceptibility of the spheroid model to doxorubicin assault.

**Figure 8:**
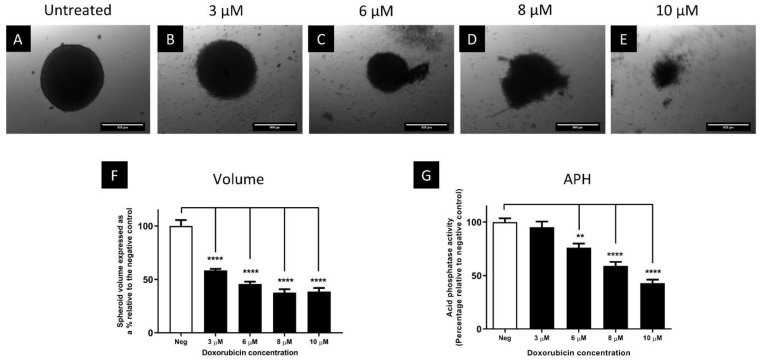
Phase contrast microscopy micrographs of spheroids treated with various concentrations of doxorubicin for 72 h (**A**–**E**). Images captured using a 5× objective lens. Scale bar = 500 µm. Panels (**F**) and (**G**) show the effect of these concentrations of doxorubicin on the volume of spheroids and APH activity of spheroids, respectively. ** = *p* ≤ 0.01, **** = *p* ≤ 0.0001. Neg = negative control.

To investigate whether BT-20 spheroids were at all sensitive to doxorubicin, spheroids were exposed to 3 to 10 µM doxorubicin for 72 h. While 1580 nM only resulted in a slight reduction, 3, 6, 8 and 10 µM doxorubicin significantly (*p* ≤ 0.0001) decreased the volume by 42, 54, 38, and 39%, respectively (Figure 8F). At high concentrations, the structural integrity of spheroids was disrupted with a loss of the smooth edges at 3 µM (Figure 8B), and disintegration of cells at concentrations higher than 6 µM (Figure 8C–E). Interestingly, the APH activity showed no reduction in activity in spheroids treated with 3 µM (*p* > 0.05) but activity was significantly (*p* ≤ 0.0001) reduced by 24%, 41% and 57% at 6, 8 and 10 µM (Figure 8G). Thus, it is suggested that BT-20 cells grown in spheroids are not completely resistant to doxorubicin, but rather withstood higher concentrations than cells grown in monolayers.

## 4. Discussion

Breast cancer is a leading cause of mortality worldwide and has recently overtaken lung cancer as the most commonly diagnosed cancer [1]. The TNBC subtype is associated with an aggressive nature, and high incidence of relapse after treatment with currently used chemotherapy regimens [2]. Thus, there is a need to investigate the biological attributes contributing to this resistance and subsequently develop novel, and effective chemotherapeutic strategies.

Traditionally, two-dimensional cell culture models have been used for pre-clinical in vitro studies [23]. However, two-dimensional culture models are associated with various limitations, which can be partially circumvented when three-dimensional culture models such as spheroids are used [11]. For example, in two-dimensional culture, all cells are exposed equally to high amounts of nutrients in media, which allows them to be synchronized in the same stage of the cell cycle [11]. Additionally, all cells cultured in two-dimensional format are exposed to the same drug concentrations of cytotoxic drugs, as the penetrative barrier that exists in in vivo settings is not recapitulated. Consequently, all cells are susceptible to the antineoplastic effects of drugs, making it appear as if the administered compound was successful. On the contrary, the third dimension in multicellular spheroids confers a barrier to penetration of drugs and nutrients. Additionally, cells are in a heterogeneous proliferative state, such as those of the in vivo tumor microenvironment. Cells in a three-dimensional culture model therefore have differential susceptibility to drug treatment, which may lead to resistance [24]. Although spheroids are used in various experimental settings, characterization thereof is presently still lacking [25]. This study was conducted to investigate the growth characteristics of BT-20 TNBC breast cancer spheroids and comparatively evaluate the cytotoxicity of doxorubicin in comparison to monolayers.

The data shows that BT-20 spheroids that were cultured using the static liquid overlay method, compacted during growth. Spheroid compaction over time is known to be a consequence of the establishment of strong intercellular connections [26]. A further decrease in the size of spheroids on subsequent days following initial formation has previously been reported by Kyfiin et al. [27]. This phenomenon is regulated by the cytoskeleton and constituents of the extracellular matrix (ECM), such as cadherins and integrins [27]. Botes et al. [15] reported that calcium-dependent cadherin adhesions play a critical role in the assembly of BT-20 spheroids. Consistent with this, strong cell–cell (cadherin) interactions were observed within the BT-20 spheroids presented here. Strong cell–cell junctions are a classic hallmark of multicellular spheroids that render them a better in vivo mimetic cell culturing model compared to two-dimensional monolayer counterparts [28].

Additionally, the BT-20 spheroids have a heterogeneous spatial cellular architecture. The spheroids have a central quiescent or necrotic region, surrounded by an outer rim of actively proliferating cells. Consistent with this, it has been reported that a necrotic core is observed in spheroids with diameters >500 µm [29]. The inner regions of the spheroid appear to be hypoxic; however, hypoxia is absent in the outer spheroid regions. Necrotic cell death can occur because of hypoxic conditions [30], therefore, the development of inner necrotic regions in this model may be linked to hypoxia. The development of hypoxia within in vivo tumors decreases the chemosensitivity of neoplastic cells [31], probably due to reduced cellular proliferation in the hypoxic zone. Furthermore, experimental hypoxia is known to induce drug resistance of various cell lines to anticancer drugs [32]. Cells in multicellular spheroids adapt to hypoxia by expressing phenotypic features that could promote survival and drug resistance [33]. Hypoxia causes replication stress, which results in the activation of the DNA damage response (DDR) kinases, Ataxia Telangiectasia Mutated (ATM) and ATM-and-Rad3-related kinases [34]. These kinases then phosphorylate downstream targets, which in turn mediate the stabilization of replication forks, preventing further DNA damage, initiating repair, and ensuring cellular survival [34]. Taking this into account, the authors hypothesized that BT-20 spheroids would confer a reduced susceptibility to anti-cancer agents compared to two-dimensional monolayers of the same cell line. To evaluate this, the in vitro cytotoxic efficacy of doxorubicin was compared in monolayers and spheroid cultures.

The IC_50_ in monolayer cultures was 310 nM; however, the cytotoxic effects of doxorubicin in three-dimensional spheroids were observed only at concentrations >3 µM (~10-fold higher concentration). Therefore, the cytotoxicity of doxorubicin was found to be attenuated in spheroids when compared to monolayers. In addition to hypoxia, various inherent phenotypic attributes of spheroids could have contributed to this reduced chemosensitivity. A study by Imamura et al. [23] found that cell lines which formed loose spheroids displayed similar sensitivity to doxorubicin to monolayers of the same cell line, while dense spheroids indicated higher drug resistance [23]. Moreover, the addition of hyaluronidase to promote penetration has been shown to enhance the cytotoxic efficacy of doxorubicin in multicellular spheroids [35]. Therefore, strong adherens junctions, which resulted in the formation of dense and compact spheroids in the current study, may have hampered the complete penetration of doxorubicin into all spheroid regions, leading to increased chemoresistance.

Enhanced cell-ECM interactions in the spheroids have recently been shown to play a pivotal role in the resistance of spheroids to doxorubicin compared to monolayers [36]. The substantial deterioration of the three-dimensional architecture of breast cancer multicellular spheroids treated with 10 µM doxorubicin was also reported [36]. Spheroid disintegration at high concentrations is most likely due to the loss of cell–cell adhesion, as doxorubicin has been shown to alter cell membrane properties [37], including E-cadherin expression levels [38]. In addition, in a previous study, novel non-sulphamoylated 2-methoxyestradiol derivatives were found to disrupt cell-substrate adhesion in BT-20 monolayers while failing to disrupt adherens interactions in spheroids at the same concentration [15]. Similarly, it is likely that while the IC_50_ of doxorubicin resulted in the detachment of cells grown as monolayers from the plastic culture plate surface, higher concentrations are required to disrupt the cell–cell adhesions of spheroids.

The observed spatial heterogeneity of cells in the spheroids could also have played a role in the reduced efficacy of doxorubicin. Doxorubicin and many other anti-neoplastic drugs target actively proliferating cells [39]. The outer, faster proliferating layer of cells of the spheroids appeared to be chemosensitive to doxorubicin, as evidenced by the decrease in spheroid size at 1580 nM, while slower-growing cells closer to the spheroid core were less sensitive. Consistent with this, doxorubicin has been found ineffective in targeting solitary dormant cells injected into a murine mouse model [40]. In addition, it has previously been reported that the necrotic areas in multicellular spheroids mimic gene expression profiles of in vivo tumors and are associated with increased drug resistance [41]. Thus, the development of necrosis in spheroids in the current study could have contributed to reduced drug sensitivity.

Additionally, Chandrasekaran et al. [42] demonstrated that BT-20 spheroids express a higher level of death receptors (DR5) and are more resistant to tumor necrosis factor-α receptor apoptosis inducing ligand (TRAIL)-mediated apoptosis, compared to monolayer cultures. Considering that doxorubicin partially exerts its cytotoxic effects through stabilization of the DR5-TRAIL apoptotic complex [43], the increased expression of DR5 could lead to circumvention of doxorubicin-induced apoptosis, and consequential reduction of the chemotherapeutic sensitivity of BT-20 spheroids.

## 5. Study Limitations

Despite the focus on a TNBC cell line in the current study, the behavior of spheroids form different hormonal subtypes under the same setting can be distinct. To further elucidate the biochemical and chemo-sensitivity characteristics that are exclusive to the TNBC subtype, other hormone-dependent breast cancer cell lines should be assessed alongside BT-20 cells. Additionally, in-depth mechanistic evaluation was not conducted to determine the specific mechanisms that can be attributed to altered chemosensitivity. Further exploration into the mechanisms of chemotherapeutic response of TNBC could play a significant role in the improvement of the clinical outcomes of TNBC, a disease associated with high relapse rates compared to other subtypes of breast cancer [44].

## 6. Conclusions

In comparison to two-dimensional monolayer cultures, the BT-20 spheroid model displayed superior in vivo mimetic attributes with respect to strong intercellular interactions and a heterogeneous spatial cytoarchitecture. Additionally, the spheroid showed reduced susceptibility to doxorubicin in comparison to monolayers, and therefore, has the potential to be used as a valuable platform for the in vitro evaluation of strategies to circumvent drug resistance in TNBC. However, further investigation into the dynamics of spheroid formation, mechanisms underlying chemosensitivity, and the effect of a wider panel of antineoplastic drugs is required to further characterize the model. This study emphasizes the need to characterize the basic attributes of three-dimensional cell culture models before using them as reliable pre-clinical research tools.

## Data Availability

All data created or analyzed during this study are available from the corresponding author upon request.

## Supplementary Materials

The following supporting information can be downloaded at: https://www.mdpi.com/article/10.3390/biomedicines11051484/s1, Figure S1: Dose–response curves showing the alteration of cell density, N = 5 biological repeats (A) and APH, N = 4 biological repeats (B) of monolayers treated with half-log dilutions of 32 µM doxorubicin for 72 h. The IC50 was calculated using a non-linear regression curve fit (log[inhibitor] vs. response) with a robust fit.
