# Supplementary material for: Comparative Evaluation of the Cytotoxicity of Doxorubicin in BT-20 Triple-Negative Breast Carcinoma Monolayer and Spheroid Cultures
Source: Biomedicines. 2023 May 19;11(5):1484. doi: 10.3390/biomedicines11051484 (PMC10216410; doi:10.3390/biomedicines11051484)
Supplement: Supplementary file 1 [file biomedicines-11-01484-s001.zip › biomedicines-2318251-supplementary.pdf]

Supplementary data

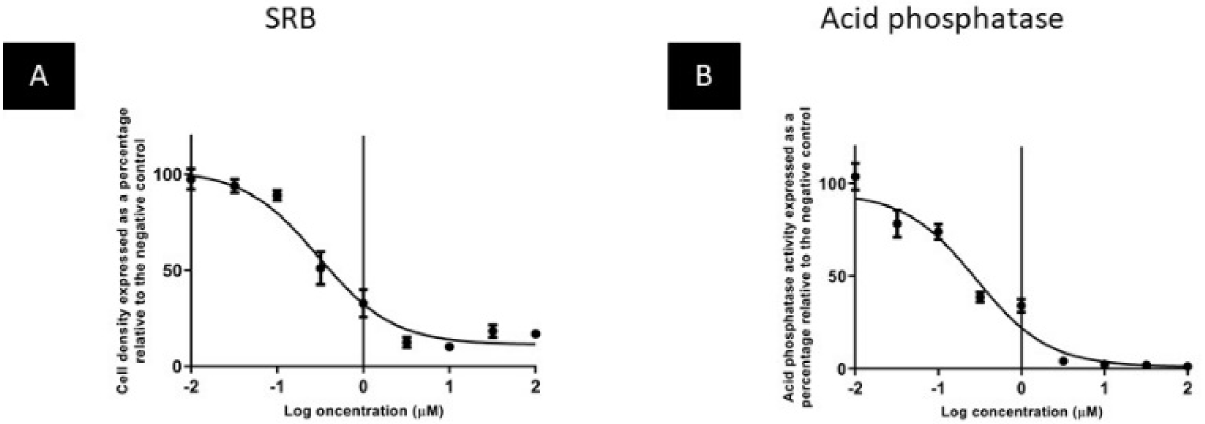

Figure S1: *Dose–response curves showing the alteration of cell density, N = 5 biological repeats (A) and APH, N = 4 biological repeats (B) of monolayers treated with half-log dilutions of 32 µM doxorubicin for 72 h. The* IC_{50} *was calculated using a non-linear regression curve fit (log[inhibitor] vs response) with a robust fit.*
